# 174. Impact of Ceftaroline Duration after Bacteremia Clearance When Used in Combination Therapy for Persistent or High-grade MRSA Bacteremia

**DOI:** 10.1093/ofid/ofad500.247

**Published:** 2023-11-27

**Authors:** M Gabriela Cabanilla, Liana Atallah, Michael Bernauer, Matthew Briski, Jason Koury, Cecilia Thompson, Chelsea Rodriguez, Bernadette Jakeman, Thomas Byrd

**Affiliations:** University of New Mexico Hospitals, Albuquerque, New Mexico; Keck Medicine of USC, Granada Hills, California; Independent Researcher, Albuquerque, New Mexico; University of New Mexico Health Sciences Center, Albuquerque, New Mexico; University of New Mexico Health Sciences Center, Albuquerque, New Mexico; University of New Mexico Health Sciences Center, Albuquerque, New Mexico; University of New Mexico Health Sciences Center, Albuquerque, New Mexico; University of New Mexico, Albuquerque, New Mexico; University of New Mexico Health Sciences Center, Albuquerque, New Mexico

## Abstract

**Background:**

Methicillin resistant Staphylococcus aureus (MRSA) bacteremia is a clinically relevant syndrome with high mortality. Combination therapy with beta-lactam antibiotics has been employed in persistent MRSA bacteremia with reported clinical success. Ceftaroline is a beta-lactam antibiotic with activity against MRSA, which has been commonly used in combination therapy for this indication. However, adequate ceftaroline duration after bacteremia clearance continues to be elucidated. Thus, we aimed to evaluate clinical outcomes of patients receiving short vs. long duration of ceftaroline after persistent or high-grade bacteremia clearance.

**Methods:**

This was a single-center, retrospective cohort study conducted from January 1, 2014, to June 30, 2021. Patients were included if ≥18 years of age, with high grade or persistent MRSA bacteremia, who received combination therapy with ceftaroline. The primary outcome was 30-day mortality. Secondary outcomes included hospital length of stay, time to bacteremia clearance, antibiotic-related adverse events and microbiological relapse.

**Results:**

A total of 32 patients were included (mean age: 51.5 years; 71.9% white; 61.3% male). 96.9% had high-grade MRSA bacteremia and 68.8% had persistent bacteremia. The most common bacteremia source was bone and joint infections (28.1%). 56.3% of patients received ceftaroline in combination with vancomycin; mean duration was 8 days. Multivariable analysis showed increasing the duration of combination therapy after bacteremia clearance was not associated with a lower risk of 30-day mortality after controlling for Charlson comorbidity index and Pitt bacteremia scores (OR 0.75, 95% CI 0.94-2.45; p=0.07). Similarly, no association was observed with microbiological relapse (OR 1.03, 95% CI 0.92-1.16; p=0.73), antibiotic-related adverse event (OR 0.97, 95% CI 0.78-1.10; p=0.75), time to bacteremia clearance (p=0.88) or hospital length of stay (p=0.06).

**Conclusion:**

Longer durations of ceftaroline in combination therapy after bacteremia clearance was not associated with lower rates of 30-day mortality, microbiological relapse, or higher rates of antibiotic-related adverse events. Larger studies are needed to determine the appropriate duration of combination therapy.

**Disclosures:**

**Cecilia Thompson, PhD, D(ABMM), MLS (ASCP)**, Roche Diagnostics: Honoraria

